# Mitigation of Sink Voids in Thick-Walled Thermoplastic Components via Integrated Taguchi DOE and CAE Simulations

**DOI:** 10.3390/polym17081126

**Published:** 2025-04-21

**Authors:** Feng Wang, Wenbo Luo, Jiling Bu, Bo Zou, Xingwu Ding

**Affiliations:** 1 College of Mechanical Engineering and Mechanics, Xiangtan University, Xiangtan 411105, China; wangfeng4@csrzic.com; 2 Zhuzhou Time New Material Technology Co., Ltd., Zhuzhou 412007, China; bujiling@csrzic.com (J.B.); zoubo@csrzic.com (B.Z.); dinghw@csrzic.com (X.D.); 3 School of Civil Engineering, Changsha University, Changsha 410022, China

**Keywords:** gauge plate, sink void, Taguchi technique, volume shrinkage, frozen layer fraction, temperature distribution

## Abstract

A gauge plate is a typical thick-walled injection-molded component featuring a complex construction used in high-speed railways, and it is prone to sink voids during the injection process. It is difficult to obtain a void-free injection molded part due to uneven cooling-induced localized thermal gradients, crystallization shrinkage of semicrystalline thermoplastics, fiber orientation-induced anisotropic shrinkage, injection parameter-dependent fountain flow, and inconsistent core compensation. This work employed design of experiment (DOE) and computer-aided engineering (CAE) simulations to analyze the influence of injection parameters on the volumetric shrinkage of the gauge plate and to identify the optimal injection process. A Taguchi orthogonal array L9 was applied, in which four injection molding process parameters were varied at three different levels. The fundamental causes of sink void defects in the gauge plate were then examined via MoldFlow analysis on the basis of the optimized injection parameters. The MoldFlow study indicates a high probability of the presence of sink void defects in the injection-molded gauge plate. To minimize sink void defects, a structural optimization design of the gauge plate was implemented to achieve a more uniform wall thickness, and the advantages of this optimization were demonstrated via comparative analysis. The small batch production of the injection-molded gauge plates demonstrates that the optimized gauge plate shows no sink voids, ensuring consistent quality that adheres to the engineering process and technical specifications.

## 1. Introduction

The growing development of high-speed railways has necessitated enhanced specifications for railway fastening systems. The gauge plate is one of the typical load-bearing components used to ensure the stability of the railway in the fastening system; it receives pressure from the elastic bar and fatigue loads from high-speed trains, subsequently transferring these loads to the underlying foundations, such as track plates or sleepers. The gauge plate is usually manufactured via an injection molding method. Nevertheless, it is typically thick-walled, rendering it susceptible to the formation of sink voids during the injection process. The existence of sink voids does not impact the short-term performance of the plate; however, prolonged service leads to material aging, resulting in a significant deterioration in the mechanical properties of the material near the sink voids due to the coupled stress concentration, ultimately reducing the service life [1,2,3,4,5,6,7,8,9].

Injection molding is a widely utilized method for processing polymers. It is a versatile technique capable of producing substantial quantities with excellent dimensional tolerance. The quality of the product depends on the material utilized, the design of the mold, and the conditions of processing. The Taguchi DOE method was employed to find the optimal values of the objective function in manufacturing operations. In contrast to traditional experimental designs, the Taguchi method employs a unique design to assess quality features with a reduced number of experiments. Comprehensive studies on the optimization of the molding process via the Taguchi method have been conducted. Chen et al. [10] integrated the Taguchi L_25_ (5^4^) orthogonal array with response surface methodology (RSM) and a hybrid GA-PSO algorithm for multi-objective optimization. The signal-to-noise (S/N) ratio and analysis of variance (ANOVA) were employed to obtain the optimal combination of process parameters to enhance the quality of length and warpage. Shen et al. [11] combined the numerical simulation with the Taguchi L_18_ (3^7^) orthogonal array to address both process conditions and cavity geometry. This holistic strategy effectively minimized sink marks by simultaneously optimizing operational and design factors. Erzurumlu et al. [12] employed the Taguchi L_18_ (3^5^) orthogonal array and ANOVA to minimize both warpage and sink index in plastic parts and determine the optimal parameter combinations, offering a streamlined method to tackle complex defect interdependencies. Another approach involves computer-aided engineering (CAE). The studies demonstrated that this approach was deemed relatively more economical, and virtual trial runs could be conducted more swiftly, producing more precise outcomes. Oktem et al. [13] applied the MoldFlow simulation by utilizing the combination of process parameters based on three levels of L_27_ (3^5^) and L_9_ Taguchi orthogonal design for virtual experimentation, along with signal-to-noise (S/N) ratio and analysis of variance (ANOVA), to optimize process parameters and reduce warpage and shrinkage defects in PC/ABS thin-shell plastic parts. Zhang et al. [14] utilized Taguchi design (L_18_ (2^1^  ×  3^7^)) and Moldflow software, along with the particle swarm optimization algorithm, to analyze and simulate the injection molding process parameters of the ABS back cover of the LCD monitor to obtain its minimum warpage.

This work aimed to enhance the quality of the gauge plate by proposing an integrated strategy that combines the Taguchi DOE method and CAE simulation. The Taguchi DOE method was employed to determine the optimal injection molding parameters to minimize volumetric shrinkage. Furthermore, MoldFlow simulation was used to investigate the origins of sink voids in the injection-molded gauge plate. Optimizing the design enhances the uniformity of the wall thickness and the process performance and reduces the risk of sink voids. The optimized design has been experimentally validated to meet the requirements of the updated fastener system standard. The findings of this study may have potential uses in molding factories and in the automotive industry.

## 2. Mechanism of Sink Voids/Sink Marks

**Figure 1:**
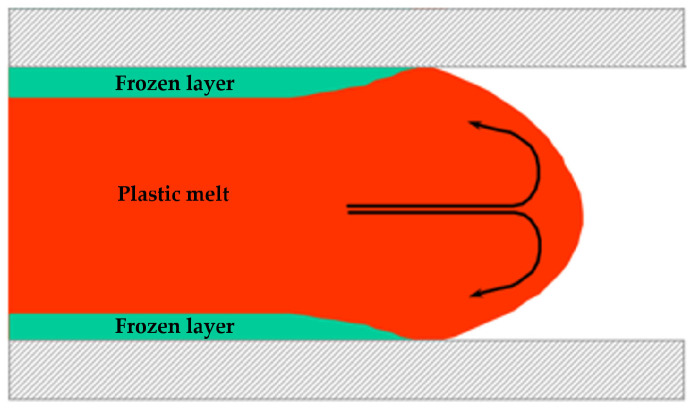
Fountain flow during the injection process.

**Figure 2:**
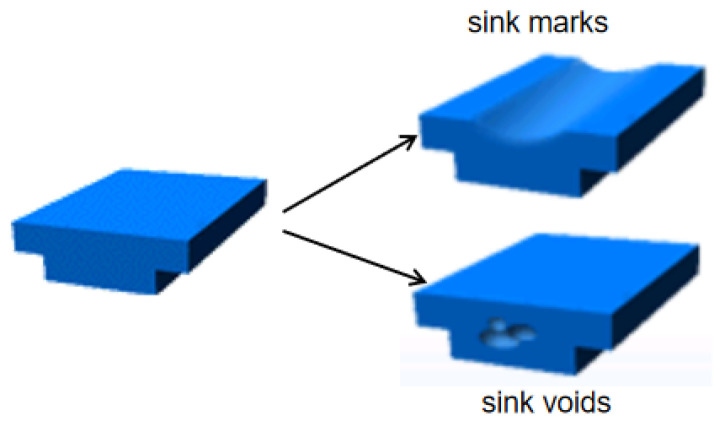
Formation mechanism of sink marks and sink voids.

Sink marks and sink voids are common flaws in thick-walled injection-molded products. Sink marks imply surface defects, indicating that the product shrinks toward its core, whereas sink voids refer to the internal cavities present within the product. The emergence of these defects is associated with the flow characteristics of the plastic melt during the filling phase of the injection molding. Typically, plastic melt fills a mold through the “fountain flow” shown in Figure 1, as described by Schmidt [15]. In this filling mode, the melt initially solidified at the surface due to direct contact with the cooler mold wall, while the core remained in a molten state. Upon completion of the filling procedure, the exterior was completely solidified, whereas the interior remained unsolidified. Inadequate pressure for prompt compensation may cause the core’s contraction to retract the skin, creating concave sink marks; conversely, if the skin is sufficiently rigid to withstand shrinkage, sink voids will develop, manifesting as vacuum bubbles, as illustrated in Figure 2.

## 3. Mechanism of Shrinkage

The formation of sink marks and sink voids is directly correlated with the shrinkage of the plastic melt. Shrinkage refers to the dimensional reduction of a plastic component when it is extracted from a mold and subsequently cooled to room temperature. The shrinkage value of a plastic component can be expressed as follows [16]:

(1) $$k=\frac{L_{m}-L_{1}}{L_{1}}\times 100\%$$

where *k* is the plastic shrinkage; $L_{m}$ are the dimensions of the mold at room temperature, mm; $L_{1}$ are the dimensions of the plastic part at room temperature, mm.

Shrinkage occurs during three phases of the injection process: the packaging phase, the cooling phase, and the demolding stage. The primary factors contributing to shrinkage include thermal shrinkage due to temperature variations, crystallization shrinkage resulting from a reduction in specific volume, orientation shrinkage of the polymer chain, compression shrinkage, and elastic recovery following the release of pressure after demolding [17,18].

### 3.1. Thermal Shrinkage

Thermal shrinkage refers to the shrinkage of a polymer melt at elevated temperatures during cooling. It is the inherent thermal physical characteristic of materials and depends on the coefficient of thermal expansion.

### 3.2. Phase Transition Shrinkage

Phase transition shrinkage refers to the shrinkage caused by a reduction in the specific volume due to the crystallization of macromolecules during the cooling stage of injection molding [19,20,21]. When the cooling temperature of the melt within the mold cavity approaches its crystallization temperature, significant changes in specific volume occur, with increased crystallinity resulting in greater shrinkage. On the other hand, higher crystallinity of the material results in increased density, a decreased linear expansion coefficient, and a reduced contraction rate. The real shrinkage rate is determined by the combined effect of these two factors. For crystalline plastics, the volumetric shrinkage caused by crystallization is much greater than the thermal shrinkage.

### 3.3. Orientation Shrinkage

Orientation shrinkage occurs due to the shear stress generated during polymer melt flow. The shear stress forces the molecular chains to orient along the flow direction [22]. A greater flow speed is correlated with a stronger molecular orientation. The forced stretching orientation of the molecular chain in the direction of flow is frozen during the cooling stage. Simultaneously, the macromolecules tend to revert to a curled state, thereby contracting in the direction of their orientation. This contraction resulting from the progressive loss of molecular orientation is associated with the intensity of the internal stress generated by the orientation process. As the internal stress increases, the contraction intensifies. Materials with a greater degree of molecular orientation demonstrate greater differences in molding shrinkage between the flow direction and its perpendicular direction.

### 3.4. Compression, Contraction, and Elastic Recovery

Thermoplastics are generally compressible, and their specific volume is altered under high pressure. At a given temperature, increasing the pressure reduces the specific volume of the material, increases the density, diminishes the expansion coefficient, and decreases the shrinkage rate. In contrast to molding shrinkage, when the injection-molded product is removed from the mold cavity, the product volume becomes larger because of elastic recovery, thus offsetting the molding shrinkage of the product to some extent [23].

## 4. Analysis of Sink Voids

### 4.1. Injection-Molded Product

**Figure 3:**
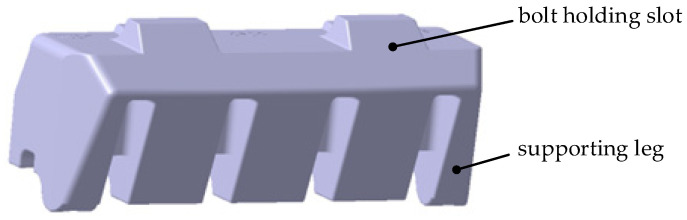
Three-dimensional profile of the gauge plate.

**Figure 4:**
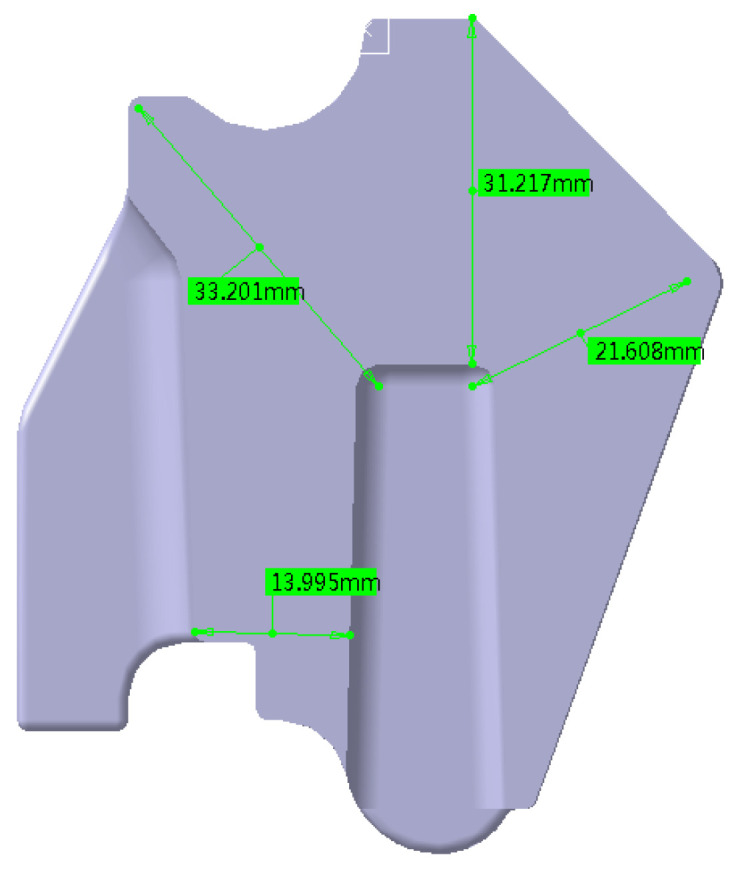
Cross-sectional dimensions of the gauge plate.

This work investigated an injection-molded gauge plate, which is a typical thick-walled composite component made of PA66-NPG30BK001 (PA66 + 30%GF). The raw materials were provided by Kingfa Science and Technology Co., Ltd., in Guangzhou, China, and dried at 120 °C for 4 h before injection molding. Figure 3 and Figure 4 show the three-dimensional profile and the primary section dimensions of the gauge plate. Thick-walled products easily cause discrepancies in the cooling rate from the surface to the core, resulting in thermal shrinkage deformation. The matrix of the gauge plate is PA66, which is a semicrystalline material; it experiences phase transition shrinkage during the cooling stage in the injection molding. Moreover, 30 wt% glass fibers are used as reinforcements, and the fibers are oriented in the direction of the melt flow together with the PA66 molecular chains, resulting in significant orientation shrinkage when cooling. Consequently, the gauge plate is extremely likely to generate sink voids during the injection molding process. Given that the gauge plate is a load-bearing structural component with specific mechanical property requirements and that sink voids adversely affect its long-term performance, it is highly practical to study the mechanism of sink void formation in the gauge plate and explore some viable strategies for minimizing these voids.

### 4.2. Design of Experiment: Taguchi Method

The majority of research on shrinkage has focused predominantly on the influence of processing parameters [11,12]. This focus arises from the necessity of numerous trials to ascertain the material properties that impact shrinkage, which is time-consuming and requires specialized expertise. The process parameters have emerged as critical determinants of product quality under specific material and mold conditions. These parameters include molding time, temperature, pressure, and others, each exerting varying and sometimes contradictory effects on shrinkage. Consequently, it is imperative to study the effects of diverse process parameters on product shrinkage and subsequently devise optimal combinations of these parameters to guarantee product quality.

The volume shrinkage qualitatively represents the linear contraction of a polymer. A smaller variation in volume shrinkage (the difference between the greatest and minimum volume shrinkage values) indicates greater uniformity in the volume shrinkage of the product. Consequently, the change in volume shrinkage is designated as a quality metric in this study.

The average volume shrinkage is defined on the basis of the relaxation density of the polymer during relaxation to ambient temperature at standard atmospheric pressure after the completion of the packing stage, as follows:

(2) $$V_{\mathrm{Shrin}}(t)=1-\frac{\bar{\rho }(t)}{\rho_{0}}$$

where $\rho_{0}$ denotes the density at ambient temperature and standard atmospheric pressure, and $\bar{\rho }$ denotes the average density along the thickness.

(3) $$\bar{\rho }(t)=(1/2h)\int_{-h}^{h}\rho (z,t)dz$$

The Taguchi method [24] was used to study the effects of the process parameters on shrinkage. It is feasible to identify the optimal processing conditions with considerable precision for reducing shrinkage. It is recognized that the significant parameters affecting the volumetric shrinkage defect in the injection molding process include mold temperature, melt temperature, injection time, packing time, packing pressure, cooling time, cooling temperature, etc. [12]. In this work, the controllable factors selected were the injection time, melt temperature, packing pressure, and packing time, designated factors A, B, C, and D, respectively. It is presumed that there is no interaction between the factors. For each factor, three levels were uniformly selected within the value range, which basically comes from literature and engineering production trials, as shown in Table 1.

The L_9_ (3^4^) orthogonal array was adopted as the test scheme, and the change in the volume shrinkage rate was designated as the quality indicator. The molding process was analyzed using MoldFlow software with version 2015. The layout of the L_9_ orthogonal array and the numerical outcomes are presented in Table 2.

**Figure 5:**
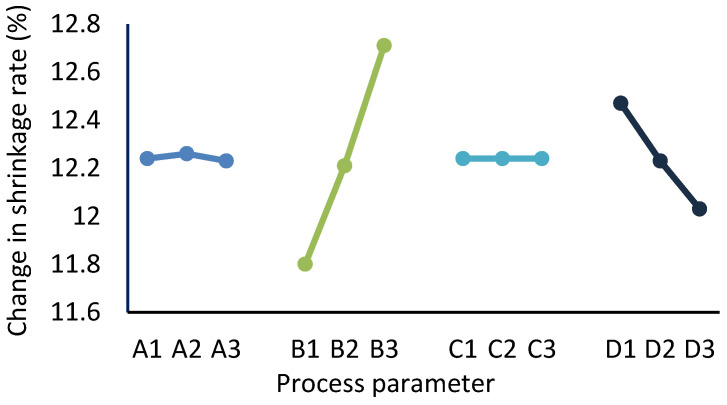
Influence of process parameters on the change in volumetric shrinkage.

The response to the change in the volume shrinkage rate is given in Table 3. As indicated by the data in Table 3, Figure 5 shows the variations in the volume shrinkage rate with the level of each factor. To minimize sink voids, the change in volume shrinkage should be minimized; therefore, the optimal combination of parameters can be identified by selecting the level with the lowest value for each factor. As a result, the optimal process parameter combination is A3, B1, C2, and D3, corresponding to an injection time of 4 s, a melt temperature of 265 °C, a packing pressure of 80 MPa, and a packing time of 70 s. This optimal parameter combination serves as the process condition for the subsequent simulation analysis and trial production.

Additionally, the impact of process parameters on shrinkage can be accessed via range values R, which represent the difference between the maximum and minimum mean values of the quality metric associated with a factor across different levels, as shown in Table 3. A higher range value indicates a stronger influence of the factor on the quality metric as the level changes. Consequently, the range value identifies the most significant factor for the shrinkage of the injected parts. According to the data in Table 3, factor B has the highest R value; consequently, the melt temperature is the most significant factor, followed by the packing time and injection time, whereas variations in the packing pressure within the established level range do not impact the shrinkage of the parts.

### 4.3. Initial CAE Simulation

**Figure 6:**
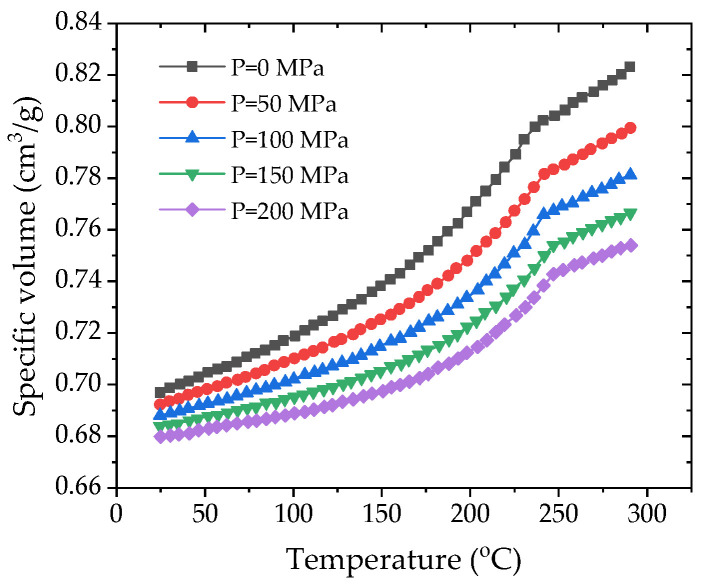
PVT curves.

**Figure 7:**
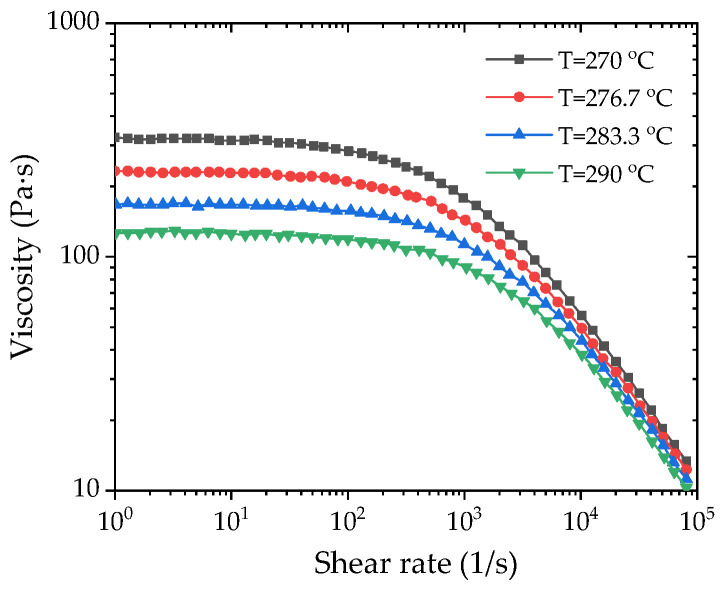
Shear viscosity curves.

**Figure 8:**
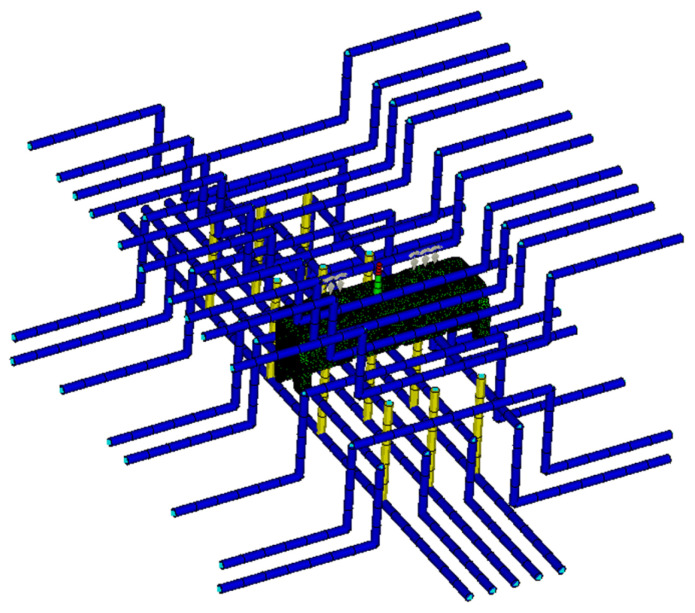
CAE simulation mesh.

The injection molding process of the gauge plate was numerically simulated via Moldflow software with version 2015 to investigate sink void formation mechanisms. The analysis employed PA66-NPG30BK001 (PA66 reinforced with 30% glass fiber, PA66 + 30GF for short), matching actual production material specifications. The properties of the material are given in Table 4, in which the PVT and shear viscosity curves are plotted in Figure 6 and Figure 7, respectively. The simulation incorporated an optimized process parameter set consisting of a 4-s injection time, a 70-s packing time under 80 MPa packing pressure, and a total cycle time of 140 s, as obtained by the Taguchi method in Section 4.2 and listed in Table 5. A needle-valve-controlled hot runner system was implemented in the finite element model, which comprehensively addressed four critical subsystems: a cooling system, a flow channel system, an exhaust system, and the final part geometry (Figure 8). For meshing, due to the complex geometry and the varied wall thickness, the 3D meshing technique was used to generate 1,764,928 elements for the analysis. The cooling system and the flow channel system were designated as beam elements.

#### 4.3.1. Frozen Layer Fraction

**Figure 9:**
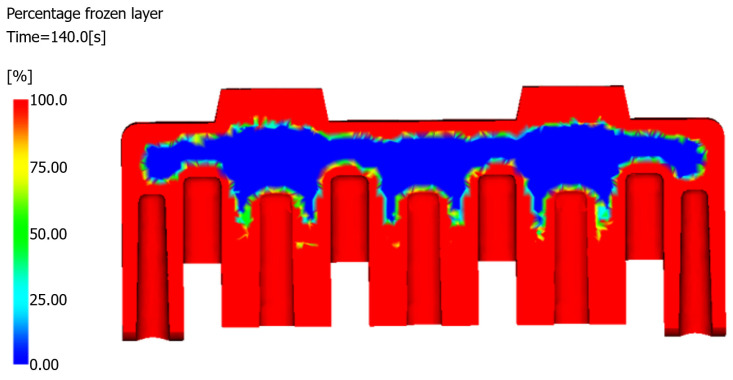
Frozen layer fraction at the end of the production cycle.

The frozen layer fraction, defined as the ratio of solidified material thickness to total wall thickness, serves as a critical indicator of the volumetric shrinkage potential. As shown in Table 6, the simulation revealed that at the completion of the packing phase (63.75 s), the frozen layer fraction reached 49.56%, indicating that 50.44% of the component’s 369 cm^3^ volume solidified without pressure compensation. This substantial unprescribed solidification creates differential shrinkage patterns that significantly increase sink void risk [25,26]. However, in real production, if the packing time is prolonged, it is difficult to seal the product within the specified time, which is not allowed. At the end of the production cycle (at 140 s), the simulation demonstrated a moderate frozen layer fraction of 75%, with residual molten material concentrated in the core region of the product (Figure 9). This thermal gradient establishes ideal conditions for sink void formation; the solidified surface layer restricts material flow, whereas the molten core continues contracting. The resulting stress concentration between the constrained surface layers and the shrinking internal material creates tensile forces that promote cavity formation.

#### 4.3.2. Temperature Distribution

**Figure 10:**
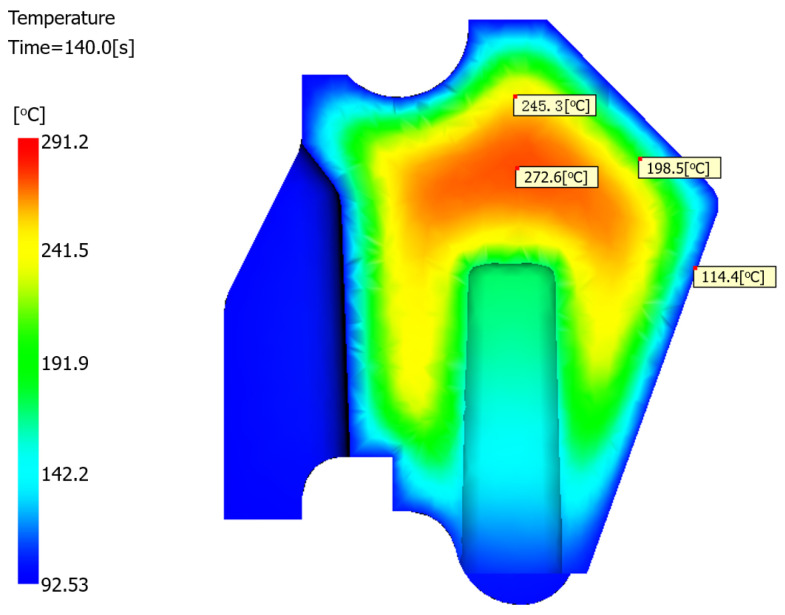
Cross-sectional temperature distribution profile.

At the conclusion of the production cycle, the mold temperature in the core region of the product reaches 272 °C, as evidenced by the temperature profile shown in Figure 10. This elevated core temperature persists while the surface material solidifies. During this freezing process, the resultant shrinkage tension is insufficient to draw the surface inward, consequently leading to the formation of sink voids within the product interior.

The above analysis of the frozen layer fraction and the temperature distribution indicates that the conventional gauge plate design presents a significant risk of sink void formation during the molding process.

### 4.4. Comparison with Actual Production Samples

**Figure 11:**
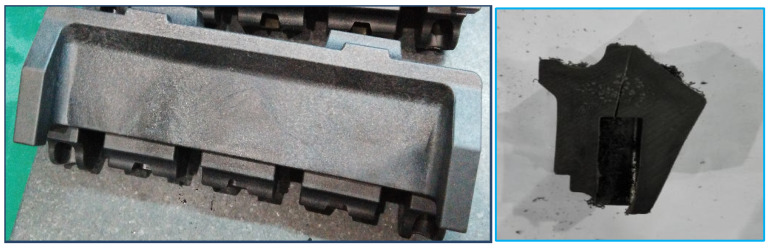
Cross-sectional view of sink void defects.

The occurrence of sink void defects is notably prevalent in actual production samples. Sectional analysis reveals substantial sink void formation, particularly in proximity to the holding slot (Figure 11). This phenomenon is attributed to the increased wall thickness in this region, as illustrated in Figure 4. The resulting disparity in solidification rates between the surface and the core layers creates favorable conditions for void formation.

## 5. Optimization Strategy and Verification

### 5.1. Design Optimization

The formation of sink voids is affected by multiple factors, including product geometry, mold configuration, processing parameters, and material properties. While various mitigation strategies have been implemented, such as process parameter optimization, cooling system enhancement, and material formulation improvements, these measures alone have proven insufficient for complete void elimination. However, the primary determinant of void formation remains the product’s structural design. For a thick-walled product, keeping the wall thickness as consistent as possible is an efficient solution. Through comprehensive structural optimization, significant improvements in void reduction have been achieved, as detailed below.

#### 5.1.1. Groove Implementation Strategy

**Figure 12:**
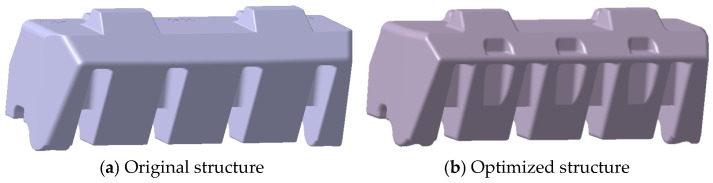
Comparative analysis of product structures—grooving implementation.

Maintaining the original installation surface geometry, strategic grooving has been implemented on the reverse side of the product to reduce the localized wall thickness (Figure 12). This modification enhances the wall thickness uniformity, thereby decreasing the propensity for void formation.

#### 5.1.2. Bottom Hole Area Enhancement

**Figure 13:**
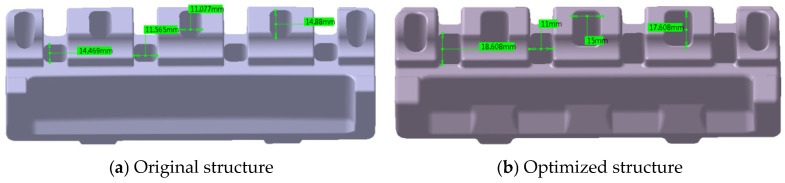
Comparative analysis of product structures—bottom hole enlargement.

The bottom hole area is strategically increased while maintaining product performance specifications (Figure 13). This modification effectively reduces the wall thickness variation, consequently minimizing the void formation potential. The preoptimization dimensions of the bottom holes are 14.47 × 11.57 mm and 11.08 × 14.88 mm, respectively, and the post-optimization dimensions are 18.61 × 11.00 mm and 15.00 × 17.61 mm, respectively.

#### 5.1.3. Hole Depth Optimization

**Figure 14:**
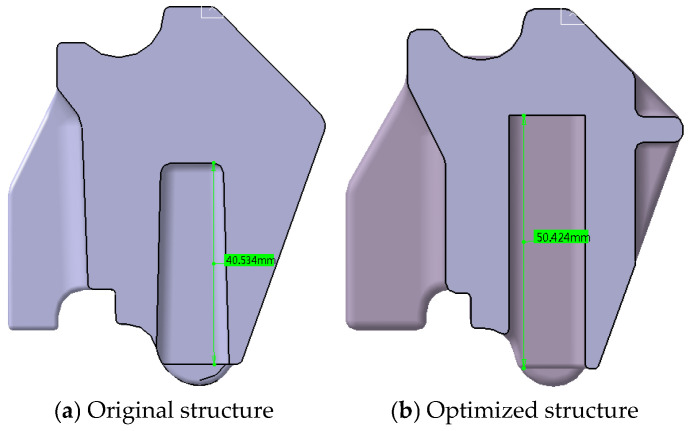
Comparative analysis of product structures—hole depth modification.

Complementing the area enhancement, the bottom hole depth increased from 40.53 mm to 50.42 mm, as shown in Figure 14. This modification further reduces localized wall thickness variation, contributing to improved product quality.

### 5.2. Further CAE Simulation

Computer-aided engineering (CAE) simulations were systematically employed to mitigate the time and financial losses associated with iterative mold corrections. The optimized design underwent rigorous process simulation, with cross-comparative analysis against the original structure, validating the feasibility of the proposed modifications. This verification enabled subsequent mold design implementation.

Identical process parameters were applied to both structural configurations to isolate performance differences. A comparative evaluation of critical metrics—including the frozen layer fraction and temperature distribution—quantitatively confirmed the enhanced performance of the optimized design.

#### 5.2.1. Frozen Layer Fraction Analysis

As summarized in Table 7, the optimized product with a volume of 348 cm3 exhibited a frozen layer fraction of 66.8% at the packing stage conclusion (63.75 s), representing a 34.8% improvement over the original structure’s 49.56%. This enhancement significantly reduces sink void risks caused by inadequate packing pressure.

**Figure 15:**
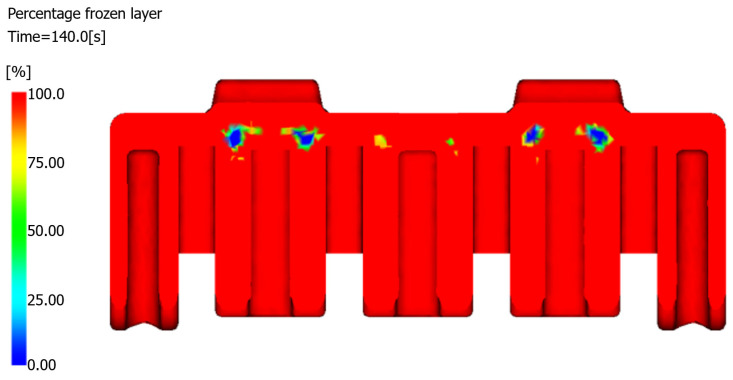
Cross-sectional frozen layer distribution of the optimized structure.

The optimized structure demonstrated accelerated solidification kinetics throughout the production cycle. At the completion of the production cycle (140 s), the frozen layer fraction reached 96.5%, whereas it reached 75.34% for the original design. The residual unfrozen material distribution is visualized in the cross-sectional frozen layer map (Figure 15).

#### 5.2.2. Temperature Profile of the Optimized Structure

**Figure 16:**
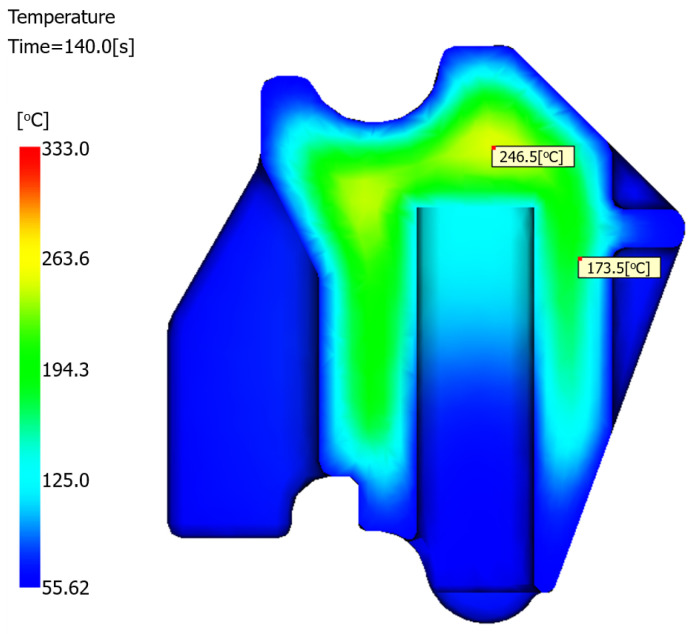
Cross-sectional temperature profile of the optimized structure.

Structural modifications improved the thermal uniformity during molding. As shown in Figure 16, for the optimized structure, the peak temperature at production cycle completion (140 s) was 246.5 °C, which decreased by 26.1 °C compared with the peak temperature of 272.6 °C in the original structure, as shown in Figure 10. This temperature reduction aligns with the established correlations between lower melt temperatures and reduced shrinkage propensity [27,28,29].

### 5.3. Practical Verification

**Figure 17:**
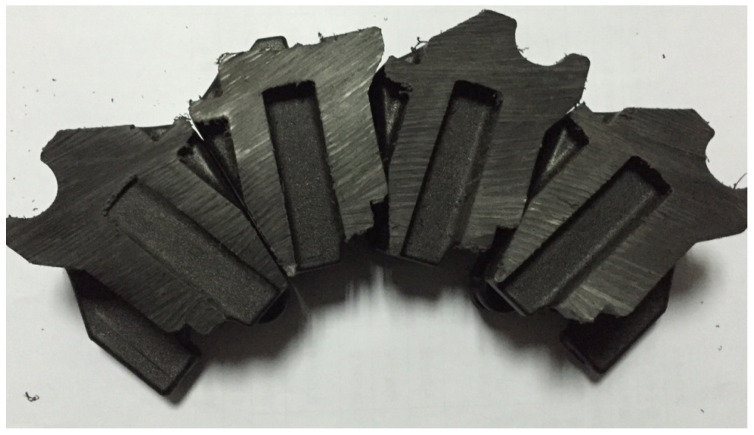
Production samples of the optimized structure.

Integrated analysis of the frozen layer fraction and temperature distribution confirmed the superiority of the optimized structure in sink void mitigation. Practical production demonstrated near-complete elimination of sink voids near the holding slot (Figure 17), corroborating the simulation predictions.

## 6. Conclusions

This study implemented a hybrid Taguchi-CAE methodology to address sink void formation in injection-molded gauge plates. The following conclusions can be drawn:

(1) The influences of four injection molding parameters on the volumetric shrinkage of the gauge plate are assessed via the Taguchi orthogonal array L9 and range values. The melt temperature was identified as the most significant factor, followed by the packing time and injection time. The optimal process parameters were as follows: melt temperature of 265 °C, injection time of 3.75 s, packing pressure of 75 MPa, and packing time of 70 s;
(2) The wall thickness variation was identified as the primary sink void formation driver, and three modifications in the structure, including backside grooving, bottom hole enlargement, and hole depth increasing, achieved thickness uniformity and reduced the risk of sink void formation;
(3) Comparative analysis of the frozen layer fraction and temperature distribution confirmed the superiority of the optimized structure design for the gauge plate. Practical production yielded void-free components, corroborating the simulation predictions and meeting all quality specifications.

## Figures and Tables

**Table 1:** Factors and levels of the orthogonal experiments.

Factor	Injection Parameters	Level 1	Level 2	Level 3
A	Injection time, A/s	3.5	3.75	4
B	Melt temperature, B/°C	265	275	285
C	Packing pressure, C/MPa	75	80	85
D	Packing time, D/s	50	60	70

**Table 2:** L_9_ (3^4^) orthogonal array and corresponding quality metric.

Experiment	A	B	C	D	Injection Time/s	Melt Temperature/°C	Packing Pressure/MPa	Packing Time/s	Change in Volume Shrinkage Rate/%
1	1	1	1	1	3.5	265	75	50	12.02
2	1	2	2	2	3.5	275	80	60	12.20
3	1	3	3	3	3.5	285	85	70	12.49
4	2	1	2	3	3.75	265	80	70	11.61
5	2	2	3	1	3.75	275	85	50	12.45
6	2	3	1	2	3.75	285	75	60	12.71
7	3	1	3	2	4	265	85	60	11.78
8	3	2	1	3	4	275	75	70	11.98
9	3	3	2	1	4	285	80	50	12.94

**Table 3:** Range analysis of the orthogonal experimental results.

Factor	Injection Time, A (s)	Melt Temperature, B (°C)	Packing Pressure, C (MPa)	Packing Time, D (s)
K1	12.24	11.8	12.24	12.47
K2	12.26	12.21	12.24	12.23
K3	12.23	12.71	12.24	12.03
R	0.03	0.91	0	0.44
Ranking	3	1	4	2

**Table 4:** The properties of material used.

Property	Value
Melt density (g/cm^3^)	1.2212
Solid density (g/cm^3^)	1.4388
Ejection temperature (°C)	215
Absolute maximum melt temperature (°C)	300
Mold temperature (°C)	75
Melt temperature (°C)	265
PVT properties	Plotted in Figure 6
Rheological properties	Plotted in Figure 7

**Table 5:** Injection parameters in Moldflow analysis.

Injection Parameter	Value
Injection time (s)	4
V/P switch-over (%)	98
Packing time (s)	70
Packing pressure (MPa)	80
Circle time (s)	140

**Table 6:** Simulation outputs at two different times.

Time (s)	Packing (%)	Injection Press (MPa)	Clamp Force (Ton)	Part Mass (g)	Frozen Layer Fraction (%)
63.75	44.01	80	79.3	492	49.56
140.00	100.00	0	0.00105	476	75.34

**Table 7:** Frozen layer fractions of the structure before and after optimization.

Time	Original Structure	Optimized Structure
3.75 s	7.2%	10.3%
10 s	19.4%	23.7%
20 s	28.5%	36.1%
40 s	40.4%	53.1%
63.75 s	49.5%	66.8%
80 s	59.7%	80.2%
120 s	70.7%	93.5%
140 s	75.34	96.5%

## Data Availability

The original contributions presented in this study are included in the article. Further inquiries can be directed to the corresponding author.
